# Insights into the role of immunosenescence during varicella zoster virus infection (shingles) in the aging cell model

**DOI:** 10.18632/oncotarget.6117

**Published:** 2015-10-14

**Authors:** Ji-Ae Kim, Seul-Ki Park, Mukesh Kumar, Chan-Hee Lee, Ok Sarah Shin

**Affiliations:** ^1^ Department of Biomedical Sciences, College of Medicine, Korea University, Seoul, Republic of Korea; ^2^ Department of Tropical Medicine, Medical Microbiology and Pharmacology, Pacific Center for Emerging Infectious Diseases Research, John A. Burns School of Medicine, University of Hawaii at Manoa, Honolulu, HI, USA; ^3^ Department of Microbiology, Chungbuk National University, Cheongju, Republic of Korea; ^4^ Department of Microbiology, College of Medicine, Korea University, Seoul, Republic of Korea

**Keywords:** immunosenescence, progeria, VZV, STING, shingles, Gerotarget

## Abstract

Varicella zoster virus (VZV) is the etiological agent of shingles, a painful skin rash that affects a significant proportion of the elderly population. In the present study, we used two aging cell models, Hutchinson-Gilford progeria syndrome (HGPS) fibroblasts and stress or replicative senescence-induced normal human dermal fibroblasts (NHDFs), to investigate age-associated susceptibility to VZV infection. VZV infectivity titers were significantly associated with donor age in HGPS fibroblasts and senescence induction in NHDFs. High throughput RNA-sequencing (RNA-seq) analysis was performed to assess global and dynamic changes in the host transcriptomes of VZV-infected aging cells. Analysis of differentially expressed genes (DEGs) indicated that VZV infection in aged HGPS fibroblasts resembled that in senescent NHDFs, particularly in terms of genes associated with pattern recognition receptors in virus sensing network, providing novel insights into the mechanisms of senescence-associated susceptibility to VZV infection. Additionally, we identified stimulator of interferon genes (STING) as a potential VZV sensing receptor. Knockdown of STING expression resulted in increased viral replication in primary fibroblasts, whereas STING overexpression led to suppression of VZV plaque formation. In conclusion, our findings highlight the important role of immunosenescence following VZV infection and provide significant insights into the mechanisms underlying cellular sensing of VZV infection and the induction of immune responses in aged skin cells.

## INTRODUCTION

Varicella zoster virus (VZV), a member of the *Alphaherpesvirinae* subfamily, causes chickenpox (varicella) upon primary infection. Reactivation of VZV from a neuronal latent state results in shingles (herpes zoster) [1]. VZV tropism is mainly associated with the skin and mucosa. In chickenpox and shingles, VZV replication in the epidermal layer of the skin results in the formation of large polykaryocytes and the development of blisters containing infectious virus. Elderly individuals exhibit increased susceptibility to shingles. The mechanisms underlying VZV susceptibility among the aged are currently unclear; however, there is some evidence to suggest that aging is correlated with dysfunctional immune cell-mediated virus clearance [2].

The efficacy and effectiveness of vaccines against VZV decrease with the age of the individual, because of the negative impact of aging on the immune system and its ability to function [3, 4]. Aging is thought to be promoted by cellular senescence, with senescent cells accumulating in the tissues and organs of aging individuals [5]. The term ‘immunosenescence’ has been used to describe the gradual deterioration of the immune system during aging. Immunosenescence appears to result in an increased susceptibility to infectious diseases and inflammation-related pathological conditions [6]. In the elderly, increased susceptibility to herpes zoster and decreased vaccine efficacy are attributed to immunosenescence [7]. The roles and mechanisms of immunosenescence in the elderly during VZV infection are yet to be fully elucidated.

Hutchinson-Gilford progeria syndrome (HGPS) is a rare genetic condition. Affected individuals have an average life span of 13 years [8]. Recent studies demonstrate that, similar to certain aspects of premature aging [9], the signaling pathway activation state in cells derived from chronologically young patients with HGPS strongly resembles that in cells from normal middle-aged and elderly individuals [10]. A silent point mutation (C1824T) in the *LMNA* gene results in the production of a truncated form of the lamin A/C protein, known as progerin, which accounts for the accelerated aging phenotype in HGPS. Progerin induces nuclear blebbing in HGPS cells grown in culture [11]. Specifically, primary fibroblasts from HGPS patients exhibit characteristic nuclear blebbing and punctate accumulation of progerin, as well as a reduced growth rate [12]. Cellular aging in HGPS fibroblast cultures is characterized by an initial period of hyperproliferation, followed by a rapid loss in the number of proliferating cells after several passages [13]. The susceptibility of aging progeria fibroblasts to viruses is yet to be examined.

An improved understanding of the changes underlying the progression of senescence, and of the role(s) of senescence during infection, may enable the development of therapeutic strategies for age-related pathologies and infectious diseases [14]. Recently, high throughput RNA sequencing (RNA-seq) technology has been used to profile host transcriptomes during viral infections and diseases [15-20]. The application of RNA-seq technology is potentially very useful for elucidation of the dynamics of the pathogen genome and the systemic changes in host gene expression in response to infection. This would enable the study of mechanisms underlying host susceptibility to viral pathogens during pathogenesis.

In the present study, we sought to characterize VZV replication efficiency in non-senescent and senescent fibroblasts. Additionally, we attempted to examine cell type- and age-specific mRNA profiles, in order to investigate the role and mechanisms of senescence during VZV replication, as well as the host response during VZV infection, in human primary fibroblasts. In addition, we used two models of aging, HGPS fibroblasts and replicative senescence-induced normal human dermal fibroblasts (NHDFs), to investigate age-associated susceptibility to VZV infection.

## RESULTS

### Characteristics of VZV infection in HGPS cells

**Figure 1:**
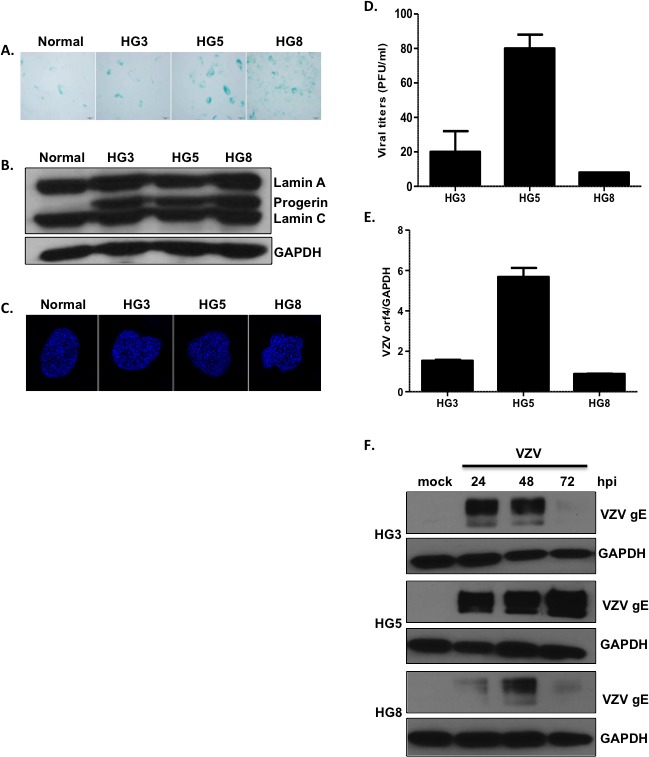
Age-associated changes in viral replication efficiency in HGPS fibroblasts (**A**) The extent of senescence-associated β-galactosidase (SA-β-Gal) staining increased with increasing age of progeria cells (HGPS cells isolated from 3- (HG3), 5- (HG5), and 8- (HG8) year-old patients). (**B**) Western blotting analysis of progerin and lamin A/C in progeria cells. Glyceraldehyde 3-phosphate dehydrogenase (GAPDH) was used as a loading control. (**C**) Progeria cell nuclei were stained with 4,6-diamidino-2-phenylindole (DAPI) and examined by confocal microscopy. Images shown are representative of results from three independent experiments. (**D**) Plaque assays were used to determine VZV titers in progeria cells. Results are presented as plaque-forming units (PFU)/mL and are the mean ± SD from three independent experiments. (**E**) We used quantitative reverse transcription polymerase chain reaction (qPCR) assays to measure the mRNA levels of VZV open reading frame 4 (ORF4; immediate early gene). Transcript expression levels were calculated in relation to the expression level of *GAPDH* mRNAs. VZV-infected HG8 cells were arbitrarily set to 1. We performed qPCR assays in duplicate, with the mean ± SD from all experiments shown. (**F**) Cells were infected with mock or VZV at a multiplicity of infection (MOI) of 0.001 for 24, 48, or 72 h, (hpi; hours post infection) and the level of VZV glycoprotein E (gE) protein present was determined by western blotting.

Progeria is a form of accelerated aging, and aged progeria fibroblasts exhibit cellular senescence [21]. Therefore, we assessed the levels of senescence in progeria fibroblasts. HGPS cells isolated from 3 (HG3)-, 5 (HG5)-, and 8 (HG8) -year-old patients were cultured and used in subsequent experiments. Senescence-associated β-galactosidase (SA-β-Gal) staining is commonly used to evaluate senescence [22]. We observed an increase in the numbers of SA-β-Gal-positive cells with increasing age of HGPS fibroblasts (Figure 1A). The levels of progerin and lamin A/C in HGPS and control fibroblasts were assessed using western blotting (Figure 1B). All HGPS fibroblasts expressed lamin A/C as well as progerin; however, progerin was not detected in the control fibroblasts. The nuclei of the control fibroblasts were round and mostly regular in size and shape. In contrast, HGPS fibroblast nuclei were more variable in size, with nuclear envelopes appearing to “bleb” near the cytoplasm, consistent with previous reports (Figure 1C) [13]. In particular, we found that the cellular growth kinetics of HG8 cells were significantly slower than those of HG3 or HG5 cells. Therefore, progeria cells at low passage (< 20) were used for all experiments.

Next, we investigated whether aged HGPS fibroblasts were more susceptible to VZV infection. HG3, HG5, and HG8 cell cultures were infected with mock or VZV, a clinical viral strain isolated from a patient with shingles [23], at a multiplicity of infection (MOI) of 0.001. Plaque assays were performed to measure viral titers. Plaque formation was significantly higher for HG5 cultures than for HG3 and HG8 cultures (Figure 1D). Consistent with these results, quantitative real-time reverse transcription polymerase chain reaction (qRT-PCR) assays revealed that the expression of the VZV gene open reading frame 4 (ORF4) was approximately 3-fold higher in HG5 cultures than in HG3 and HG8 cultures (Figure 1E). Given that glycoprotein E (gE) of VZV, encoded by ORF68, is the most abundant VZV glycoprotein [1], we measured gE protein levels in VZV-infected HGPS cells. At 24 h post-infection (hpi), gE levels were highest in HG5 cultures compared with HG3 or HG8 cultures. The expression level of gE in VZV-infected HG3 cultures peaked at 24-48 hpi, and was found to decrease dramatically at 72 hpi. The HG8 cultures exhibited lowest levels of VZV gE at all time points (Figure 1F). Based on these results, we speculated that progeria fibroblasts are susceptible to VZV infection, and that viral replication efficiency is affected by the age of cells.

### Characteristics of the replicative or stress-mediated senescence aging model and VZV replication

**Figure 2:**
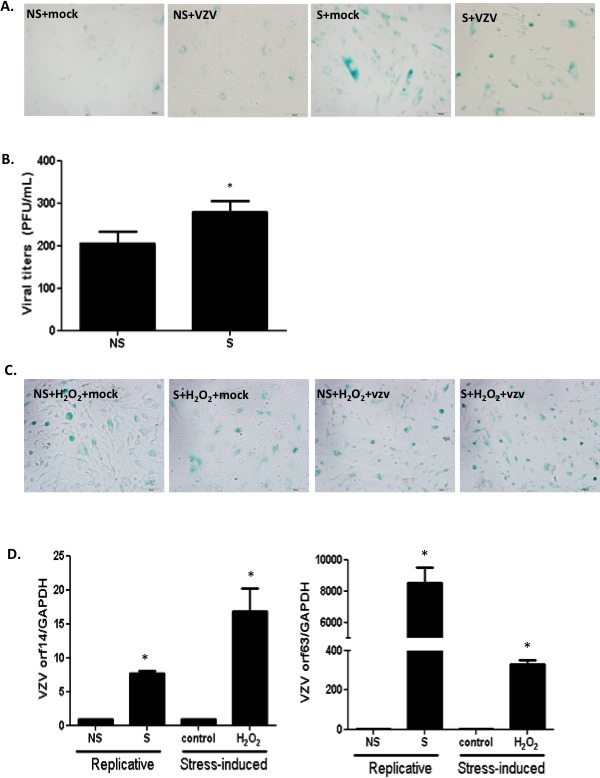
Stress-induced or replicative senescence in normal human dermal fibroblasts (NHDF) affects viral replication (**A**) Non-senescent (NS) and senescent (S) NHDFs were infected with VZV at an MOI of 0.001 and senescence-associated β-galactosidase (SA-β-Gal) was added. Senescence was induced at high passage NHDFs and confirmed by positive staining. (**B**) Plaque assays were conducted. Results are presented as PFU/mL and are the mean ± SD from three independent experiments. *p < 0.05 vs. VZV-infected NS cells. (**C**) Stress-induced senescence in hydrogen peroxide (H_2_O_2_) treated NS NHDFs was confirmed by senescence-associated β-galactosidase (SA-β-Gal) staining. (**D**) VZV ORF14 (late) and 63 (immediate early) gene expressions were measured by qPCR. Transcript expression levels were calculated in relation to the expression level of *GAPDH* mRNAs. NS cells that were VZV-infected were arbitrarily set to 1. We performed qPCR assays in duplicate, with the mean ± SD from all experiments shown. *p < 0.05 vs. mock-infected cells.

Replicative senescence was first described by Hayflick and Moorhead, who observed that human fibroblasts in culture underwent extensive replication as a consequence of serial passaging [24]. In order to confirm the senescence phenotype during replicative senescence, we compared non-senescent NHDFs at low passage with senescent NHDFs that had been subjected to over 20 passages following infection with VZV. Induction of senescence in high-passaged NHDFs was confirmed, and increased numbers of SA-β-Gal-positive cells were observed (Figure 2A). Then, we investigated whether the efficacy of VZV replication differed between non-senescent and senescent cells. Senescent NHDFs exhibited approximately 1.5-fold higher VZV infectivity titers than non-senescent NHDFs (Figure 2B). Additionally, we determined whether stress-induced senescence modulates VZV replication. Stress-induced senescence induced by H_2_O_2_ treatment of non-senescent cells was confirmed by an increase in the number of SA-β-Gal-positive cells (Figure 2C). We also measured transcriptional levels of VZV-specific genes 14 (late), and 63 (immediate early) during senescence. The mRNA levels of ORF 14 and 63 were significantly higher in VZV-infected senescent cells than in non-infected non-senescent cells (Figure 2D). Additionally, we found that oxidative stress damage due to H_2_O_2_ treatment of non-senescent cells resulted in significant upregulation of VZV gene expression, which was also observed during senescence-mediated induction of VZV replication.

### Analysis of RNA-seq transcriptome data reveals distinct and dynamic changes in aging cells

**Figure 3:**
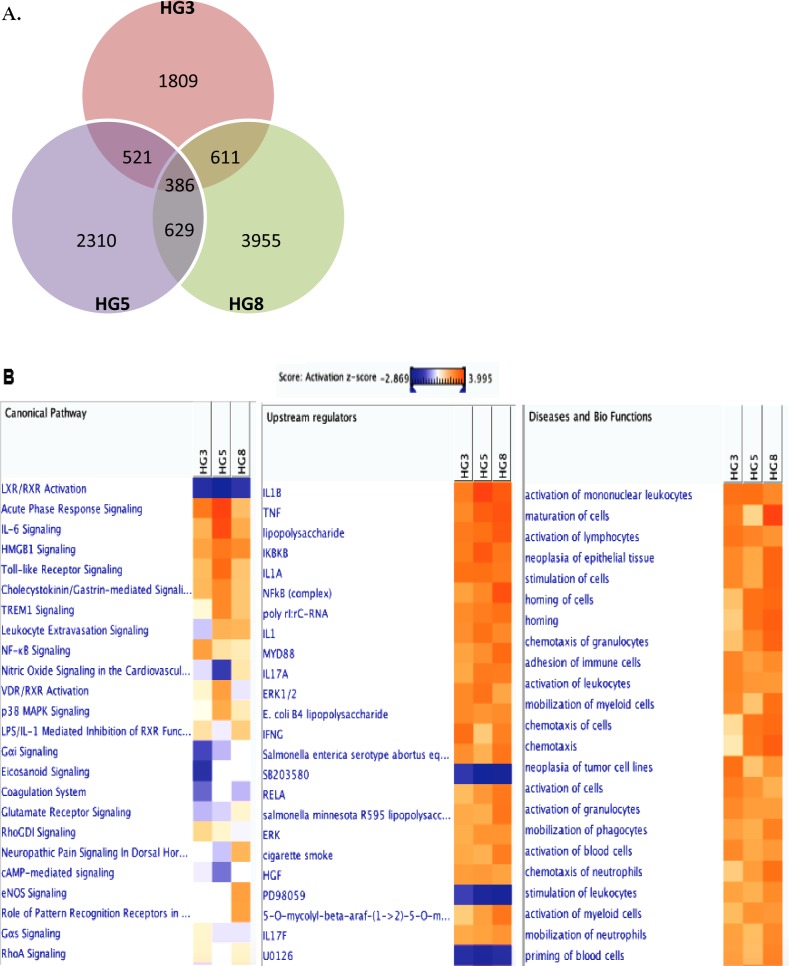
Transcriptome analysis of VZV-infected HGPS cells RNA-seq analysis was performed with RNA isolated from cells derived from HG3, HG5, and HG8 cultures that were infected with mock or VZV. (**A**) Venn diagram indicates the number of significant (>2-fold upregulation, FDR, 0.05) DEGs across three key comparisons (HG3, HG8, HG8) and the overlap between each set of genes. (**B**) Ingenuity Pathway Analysis (IPA) tool was used to generate the list of highest activated networks and genes with their respective scores obtained from IPA and categorized as canonical pathways, upstream regulators and diseases and biofunctions. Networks and genes shaded orange were upregulated, while those in blue were downregulated. Shading intensity indicates the degree that each gene was upregulated or downregulated. Range of activation z-score is also depicted on the figure.

Given than HGPS cells mimic the normal aging process at the cellular level and are susceptible to VZV infection, RNA-seq was performed to explore the transcriptome of primary fibroblasts derived from 3-, 5-, and 8-year-old HGPS patients. Primary fibroblasts were infected with mock or VZV for 24 h and RNA was isolated. More than 206 million 101-bp paired-end reads were generated. Gene expression profiles for mock-infected HG3, HG5, and HG8 cultures were compared with those of VZV-infected cultures in order to identify differentially expressed genes (DEGs) with a fold change of ± 2 and a *q*-value < 0.05. We identified 1,809, 2,310, and 3,955 upregulated genes in HG3, HG5, and HG8 cultures, respectively. Venn diagrams revealed that the cultures and VZV-infected progeria cells had 386 DEGS in common (Figure 3A). Our results showed that infection with VZV induced distinct cellular gene expression patterns in progeria cells of varying ages. In order to further characterize the DEGs, we focused on the 10 DEGs showing the most marked upregulation. Interestingly, VZV infection in HG5 fibroblasts was associated with the expression of key immune-related genes, such as members of the NOD-like receptor (NLR) family, pyrin domain-containing 3 (NLRP3) and melanoma differentiation-associated 7 (MDA7)/IL-24, whereas no immune-related genes were found among the top 10 most upregulated DEGs in HG3 and HG8 fibroblasts (Table 1). Host transcriptome data showed a higher number of upregulated DEGs associated with immune function in HG5 cells than in HG3 or HG8 cells, suggesting a strong correlation between viral transcript levels and expression levels of host immune-related genes.

**Table 1:** Top 10 Up-regulated DEG genes from VZV-infected progeria and NHDF cells

Rank	Progeria								
	HG3			HG5			HG8		
	Genes	Fold change	p value	Genes	Fold change	p value	Genes	Fold change	p value
1.	RPS4Y1	208.9535	<0.0001	CSF3	285.0072	<0.0001	C2CD4A	296.1744	<0.0001
2.	FAM19A3	168.9624	<0.0001	RPS4Y1	243.2414	<0.0001	CTD-2303H24.2	234.0324	<0.0001
3.	OSTN-AS1	168.9624	<0.0001	AC003092.1	168.0631	<0.0001	PTGDR	148.5872	<0.0001
4.	DHRS9	104.9767	0.0014	DDX3Y(	109.591	<0.0001	EHF	133.0517	0.00012
5.	GIPC2	96.97853	0.0024	FAM213A	101.2378	0.0014	GBP6	117.5162	0.00031
6.	RP11-114H23.2	88.98032	0.0041	NLRP3	101.2378	0.0014	MIR125B1	117.5162	0.00031
7.	RP3-393E18.2	80.98211	0.0041	IL24	94.47401	<0.0001	TBX5	109.7485	0.00051
8.	RP11-753N8.1	80.98211	0.0072	BTBD11 (BTB (POZ)	92.88468	0.0024	RP3-465N24.5	109.7485	0.00051
9.	ASB2	72.9839	0.0072	TRBC2	84.53153	0.0041	RDH8	101.9807	0.00085
10.	USP9Y	72.9839	0.0072	RP3-393E18.2	84.53153	0.0041	RP11-211G3.2	101.9807	0.00085

Rank	NHDF					
	Non-senescent NHDF			Senescent NHDF		
	Genes	Fold change	*p* Value	Genes	Fold change	*p* Value
1.	CXCL3	2244.541	<0.0001	CXCL8	4004.224	<0.0001
2.	CXCL8	1870.27	<0.0001	CCL7	1776.975	<0.0001
3.	MIR146A	559.8892	<0.0001	CXCL6	1511.021	<0.0001
4.	IL-1B	496.0161	<0.0001	CXCL1	774.4706	<0.0001
5.	CXCL1	474.5578	<0.0001	CCL20	663.9217	<0.0001
6.	IFI44L	388.0203	<0.0001	CSF2	508.4215	<0.0001
7.	RPS4Y1	336.3335	<0.0001	MIR146A	475.6846	<0.0001
8.	CSF2	304.397	<0.0001	RPS4Y1	385.6582	<0.0001
9.	CXCL6	298.7515	<0.0001	PTHLH	312.0003	<0.0001
10.	OASL	287.0317	<0.0001	CXCL2	264.4527	<0.0001

In order to investigate the biological interactions of DEGs and identify important functional networks, the Ingenuity Pathway Analysis (IPA) tool was applied with datasets from altered expression profiles obtained by RNA-seq analysis. Highest activated networks and upstream modulators (high z-score) were identified using IPA. Figure 3B shows separate hierarchical clustering maps depicting highly activated biofunctions, upstream regulators, and canonical pathways after VZV infection in HG3, HG5, and HG8 cells. Acute phase response signaling, Toll-like receptors (TLRs) signaling, interleukin-6 (IL-6) signaling, NF-κB signaling, and p38 mitogen-activated protein kinase (MAPK) signaling were among the highest activated canonical pathways for HG5 after VZV infection compared with HG3 or HG8 (Figure 3B). Moreover, stimulation, activation, and maturation, along with adherence and migration, were among the most highly activated biofunctions in the progeria cells following VZV infection. Consistent with the increase in senescence levels confirmed by β-galactosidase staining, HG8 cells revealed a higher z-score (3.992) for cell maturation than HG3 or HG5 cells.

**Figure 4:**
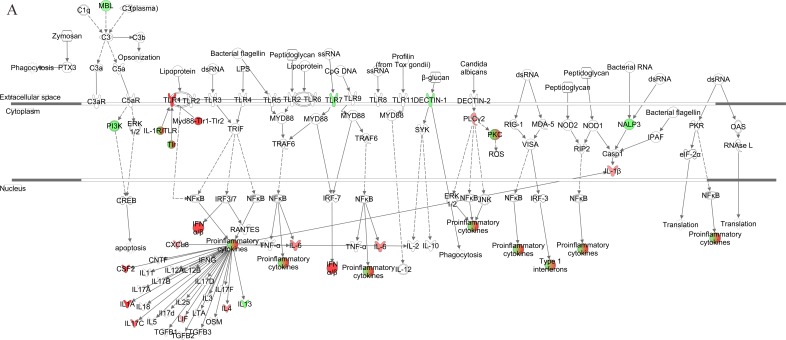

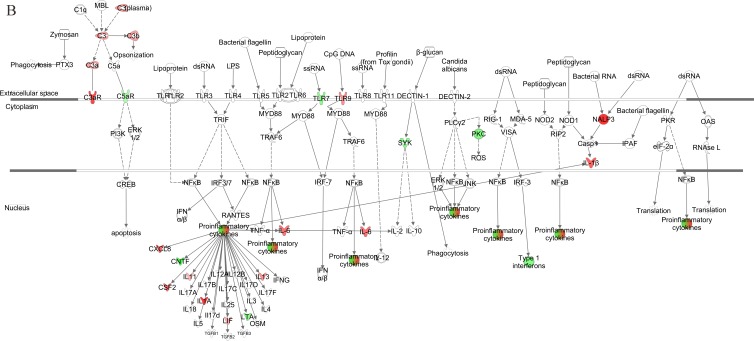
Pathway for pattern recognition receptor of VZV-infected HGPS cells Genes associated with pattern recognition receptors in virus sensing network activated by VZV-infected HG3 (**A**) and HG5 (**B**) are shown. Genes associated that show differential expression are highlighted in color. Color intensity indicates the degree of upregulation (red) or downregulation (green) relative to the mock-infected HGPS cells. Solid lines represent direct interactions and dashed lines indirect interactions.

We further generated a network map of pattern recognition signaling for HG3 and HG5 cultures infected with VZV, using the IPA tool (Figure 4). Notable differences between HG3 and HG5 cultures included differential modulation of TLR receptors, type 1 interferon, and pro-inflammatory cytokine response. Moreover, we also observed activation of NLRP3 (NALP3) in HG5 cultures, whereas the expression of NALP3 was downregulated in HG3. Furthermore, pathway analysis using the Kyoto Encyclopedia of Genes and Genomes (KEGG) revealed that the NLR signaling pathway was the most enriched pathway in VZV-infected progeria cells, consistent with IPA data (Table 2). Gene ontology (GO) classification analysis also identified distinct gene lists associated with each progeria cell, highlighting the importance of genes enriched in chemokine activity (Table 3). Collectively, our analysis of the gene expression data revealed that functions of innate receptors for viruses, IFNs, and inflammation-mediated signals were associated with the highest rated networks in VZV-infected progeria cells.

**Table 2:** The top 10 enriched KEGG pathways of DEGs in VZV-infected progeria and NHDF cells

Rank	Progeria			NHDF	
	HG3	HG5	HG8	Non-senescent	Senescent
1	Cell cycle	Cytokine-cytokine receptor interaction	Cytokine-cytokine receptor interaction	Cytokine-cytokine receptor interaction	NOD-like receptor signaling pathway
2	NOD-like receptor signaling pathway	NOD-like receptor signaling pathway	Hematopoietic cell lineage	NOD-like receptor signaling pathway	Cytokine-cytokine receptor interaction
3	Cytokine-cytokine receptor interaction	Chemokine signaling pathway	NOD-like receptor signaling pathway	Cytosolic DNA-sensing pathway	Cytosolic DNA-sensing pathway
4	Oocyte meiosis	Hematopoietic cell lineage	Pathways in cancer	Chemokine signaling pathway	Chemokine signaling pathway
5	Progesterone-mediated oocyte maturation	Complement and coagulation cascades	Jak-STAT signaling pathway	RIG-I-like receptor signaling pathway	RIG-I-like receptor signaling pathway
6	Chemokine signaling pathway	Jak-STAT signaling pathway	Apoptosis	Pathways in cancer	Hematopoietic cell lineage
7	Small cell lung cancer	Pathways in cancer	Chemokine signaling pathway	Arachidonic acid metabolism	Pathways in cancer
8	Cytosolic DNA-sensing pathway		Calcium signaling pathway	Jak-STAT signaling pathway	Bladder cancer
9	Toll-like receptor signaling pathway		Gap junction	Bladder cancer	Purine metabolism
10	p53 signaling pathway		Arachidonic acid metabolism	Hematopoietic cell lineage	Toll-like receptor signaling pathway

**Table 3:** The top 10 enriched GO molecular function terms in VZV-infected progeria cells

Rank	HGPS culture (Go terms, function		
	HG3	HG5	HG8
1	GO:0008297∼single-stranded DNA specific exodeoxyribonuclease activity	GO:0004666∼prostaglandin-endoperoxide synthase activity	GO:0005220∼inositol 1,4,5-trisphosphate-sensitive calcium-release channel activity
2	GO:0016725∼oxidoreductase activity, acting on CH or CH2 groups	GO:0003680∼AT DNA binding	GO:0004962∼endothelin receptor activity
3	GO:0016895∼exodeoxyribonuclease activity, producing 5′-phosphomonoesters	GO:0005030∼neurotrophin receptor activity	GO:0008095∼inositol-1,4,5-trisphosphate receptor activity
4	GO:0004529∼exodeoxyribonuclease activity	GO:0008330∼protein tyrosine/threonine phosphatase activity	GO:0015093∼ferrous iron transmembrane transporter activity
5	GO:0008009∼chemokine activity	GO:0043121∼neurotrophin binding	GO:0004924∼oncostatin-M receptor activity
6	GO:0042379∼chemokine receptor binding	GO:0005138∼interleukin-6 receptor binding	GO:0004571∼mannosyl-oligosaccharide 1,2-alpha-mannosidase activity
7	GO:0003777∼microtubule motor activity	GO:0004383∼guanylate cyclase activity	GO:0005138∼interleukin-6 receptor binding
8	GO:0005125∼cytokine activity	GO:0033549∼MAP kinase phosphatase activity	GO:0015924∼mannosyl-oligosaccharide mannosidase activity
9	GO:0003678∼DNA helicase activity	GO:0017017∼MAP kinase tyrosine/serine/threonine phosphatase activity	GO:0015085∼calcium ion transmembrane transporter activity
10	GO:0003697∼single-stranded DNA binding	GO:0008009∼chemokine activity	GO:0015923∼mannosidase activity

### Comprehensive analysis of functional enrichment and pathways/networks of non-senescent and senescent VZV-infected NHDFs

**Figure 5:**
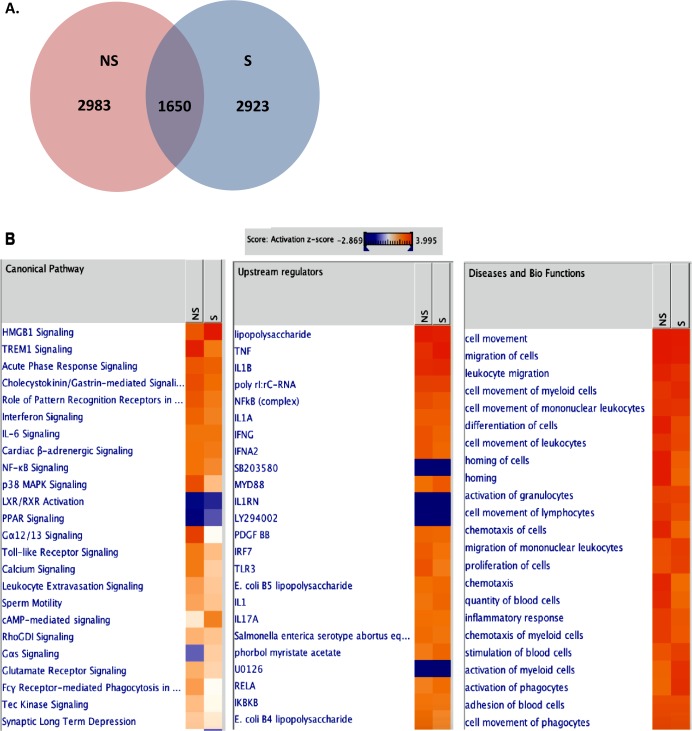
Transcriptome analysis of VZV-infected NHDFs RNA-seq analysis was performed from RNA isolation of non-senescent (NS) and senescent (S) NHDFs that were infected with mock or VZV. (**A**) Venn diagrams of overlapping significantly upregulated DEG profiles for different cell types are shown. (**B**) The IPA tool was used to generate the list of highest activated networks and genes with their respective scores obtained from IPA and categorized as canonical pathways, upstream regulators and diseases and biofunctions. Networks and genes shaded orange were upregulated, while those in blue were downregulated. Shading intensity indicates the degree that each gene was upregulated or downregulated. Range of activation z-score is also depicted on the figure.

In order to investigate whether replicative senescence induces distinct patterns of senescence-associated immune responses during VZV infection, we infected non-senescent and senescent NHDFs with VZV and performed transcriptome analysis using RNA-seq. The number of DEGs with a fold change of ± 2 and a *q*-value < 0.05 in non-senescent and senescent cells was comparable (Figure 5A). We identified 2,923 upregulated mRNAs in VZV-infected senescent cells, compared with 2,983 upregulated mRNAs in VZV-infected non-senescent cells. Venn diagrams revealed that VZV-infected senescent and non-senescent cells had 1,650 DEGs in common. In addition, chemokines, such as chemokine (C-X-C motif) ligand 8 (CXCL8), chemokine (C-C motif) ligand 7 (CCL7), and CXCL6, were among the top 10 most upregulated genes in non-senescent and senescent NHDFs infected with VZV, although expression levels of these chemokines were found to be approximately 2∼5-fold higher in senescent cells than in non-senescent cells (Table 1). IPA also identified the chemokine signaling pathway as a key pathway involving the majority of DEGs (CXCL-11, CCL7, CXCL-10, CXCL-2, and CXCL-9) across all datasets, based on the number of network interactions (Figure 5B).

Gene set enrichment analysis using KEGG identified significantly enriched functional groups according to VZV infection. Top 10 upregulated pathways activated by VZV-infected NHDFs helped identify enrichment for pathways, including the NOD-like receptor signaling pathway, cytokine-cytokine receptor interaction, cytosolic DNA-sensing pathway, chemokine signaling pathway, and RIG-I-like receptor signaling pathway (Table 2). Similar results were obtained by top 10 enriched Gene ontology (GO) analysis (Table 4). In order to investigate the possible biological interactions of DEGs, datasets representing genes with altered expression profiles were imported into the IPA tool. Highest activated networks and upstream modulators (high z-score) were identified using IPA. Figure 5B represents separate hierarchical clustering maps showing highly activated biofunctions, upstream regulators, and canonical pathways following VZV infection in non-senescent and senescent NHDFs. Acute phase response signaling, Toll-like receptors (TLRs) signaling, IL-6 signaling, NF-κB signaling, and p38 MAPK signaling were among the highest activated canonical pathways after VZV infection in NHDFs, which was consistent with observations of VZV-infected progeria fibroblasts. Canonical pathway analysis demonstrated that a higher z-score was observed for the roles of pattern recognition receptors in virus sensing and interferon signaling in non-senescent NHDFs than in senescent NHDFs.

**Table 4:** The top 10 enriched GO, molecular function terms in VZV-infected NHDF cells

Rank	Non-senescent NHDF (GO terms, function)	Senescent NHDF (GO terms, function)
1	GO:0004666∼prostaglandin-endoperoxide synthase activity	GO:0004666∼prostaglandin-endoperoxide synthase activity
2	GO:0003680∼AT DNA binding	GO:0050501∼hyaluronan synthase activity
3	GO:0004947∼bradykinin receptor activity	GO:0004947∼bradykinin receptor activity
4	GO:0005172∼vascular endothelial growth factor receptor binding	GO:0005172∼vascular endothelial growth factor receptor binding
5	GO:0008009∼chemokine activity	GO:0042910∼xenobiotic transporter activity
6	GO:0005161∼platelet-derived growth factor receptor binding	GO:0008009∼chemokine activity
7	GO:0042379∼chemokine receptor binding	GO:0042379∼chemokine receptor binding
8	GO:0002020∼protease binding	GO:0002020∼protease binding
9	GO:0005242∼inward rectifier potassium channel activity	GO:0005125∼cytokine activity
10	GO:0005125∼cytokine activity	hsa04620:Toll-like receptor signaling pathway

**Figure 6:**
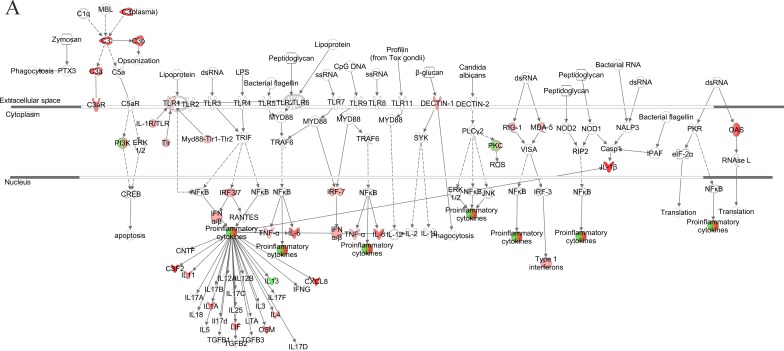

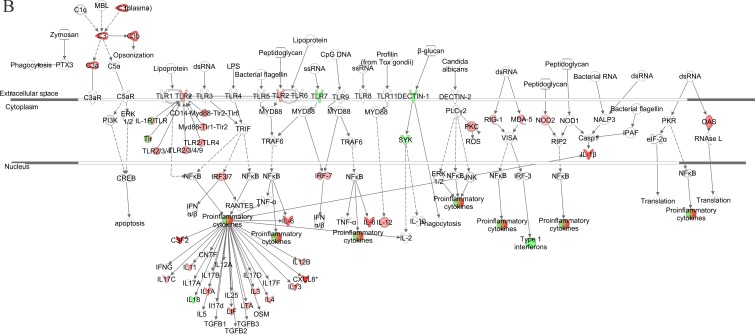
Network analysis for pattern recognition receptor of VZV-infected NHDFs Genes associated with pattern recognition receptors in virus sensing network activated by VZV-infected non-senescent (**A**) and senescent (**B**) NHDFs are shown. Genes associated that show differential expression are highlighted in color. Color intensity indicates the degree of upregulation (red) or downregulation (green) relative to the mock-infected NHDFs. Solid lines represent direct interactions and dashed lines indirect interactions.

We further generated a network map of pattern recognition signaling for non-senescent and senescent NHDFs infected with VZV (Figure 6). Similar to progeria cells, notable differences included differential modulation of TLR receptors, type 1 interferon, and pro-inflammatory cytokine response between non-senescent and senescent NHDFs. Interestingly, Type 1 interferons were upregulated to a higher extent in non-senescent cells (shown in red); however, they were found to be downregulated in senescent cells (shown in green). Collectively, the functions of innate receptors for viruses, IFNs, and inflammation-mediated signals were associated with the highest rated networks in VZV-infected NHDFs, which was consistent with findings in VZV-infected progeria fibroblasts.

### RNA-seq analysis reveals differential gene expression related to antiviral responses in aging cells

**Figure 7:**
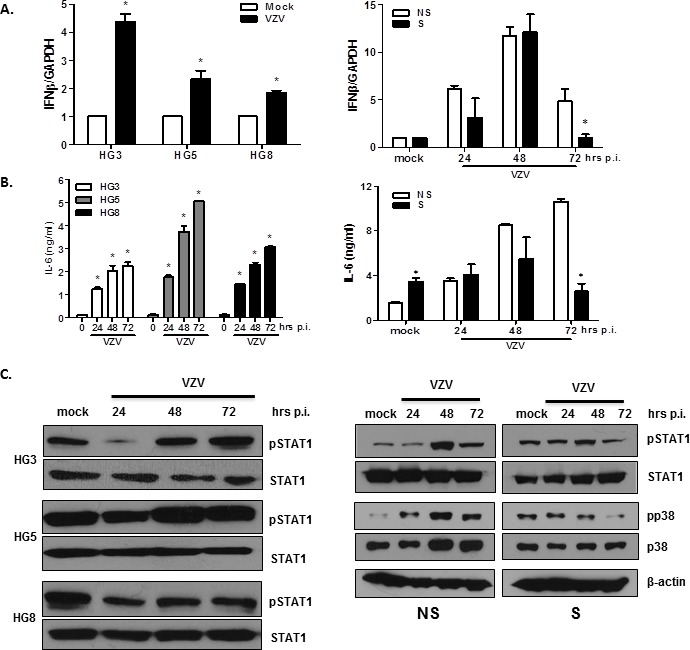
Validation of DEGs in VZV-infected non-senescent and senescent cells that are involved in antiviral responses and inflammation HGPS cells and NHDFs were infected with VZV at an MOI of 0.001 for indicated timepoints (0, 24, 48, and 72 hpi). (**A**) RNA was extracted and cDNA was made to perform qPCR. Host *IFN-β* mRNA expression levels were determined by qPCR. Expression levels were normalized to *GAPDH*. Mock-infected cells were arbitrarily set to 1. We performed qPCR assays in duplicate, with the mean ± SD from all experiments shown. *p < 0.05 vs. mock-infected cells (HGPS) and NS cells (NHDFs). (**B**) The concentration of secreted IL-6 in culture supernatants from HGPS cells and NHDFs was determined using enzyme-linked immunosorbent assays (ELISAs). Values presented are the averages from all experiments, with error bars representing SDs. *p < 0.05 vs. mock-infected cells (HGPS) and NS cells (NHDFs). (**C**) Cells were lysed and cell extracts were separated by SDS-PAGE and blotted onto nitrocellulose membranes. Protein levels of phosphorylated/total STAT1, and phosphorylated/total p38 MAPK were analyzed by western blotting. Anti- *β*-actin monoclonal antibody was used as a loading control. The images shown are representative of three independent experiments.

In order to confirm the activation of genes associated with VZV infection in the two models of cellular senescence, we used qRT-PCR assays to analyze the expression levels of selected genes involved in the IFN pathway. HGPS and NHDF cells infected with VZV exhibited significantly higher mRNA levels of IFN-β than mock-treated cells at 24 hpi (Figure 7A). Interestingly, IFN-β expression was significantly lower in HG5 and senescent NHDFs than in HG3 and non-senescent NHDFs (7.23-fold induction for non-senescent cells vs. 3.56 fold induction for senescent cells at 24 hpi). Additionally, we assessed the production of senescence-associated secretory phenotype (SASP) factors in aging cells. In both progeria and NHDF cells, IL-6 production was upregulated on VZV infection in a time-dependent manner (Figure 7B). Senescent NHDFs produced higher levels of IL-6, even in mock-infected controls, compared with non-senescent cells. These findings suggest that the induction patterns of proinflammatory cytokines/chemokines differ between non-senescent and senescent cells and correlate with viral replication efficiency.

We also investigated the activation of VZV-mediated signaling pathways involved in the interferon pathway and inflammation. Interestingly, the highest levels of phosphorylation of the signal transducers and activators of transcription (STAT1) family of transcription factors were found in HG5 cells compared with HG3 or HG8 cells, as shown in Figure 7C. Increased phospho-STAT1 (pSTAT1) expression was observed in non-senescent NHDFs at 48 hpi, in comparison with senescent NHDF cells. Induction levels of pSTAT1 correlated well with the expression levels of IFN-β in response to VZV infection. Additionally, p38 MAPK phosphorylation gradually increased over time in non-senescent cells, whereas it was suppressed at 72 hpi in senescent cells, which may have directly affected the production of proinflammatory molecules such as IL-6 (Figure 7C).

### STING as a potential receptor for VZV

**Figure 8:**
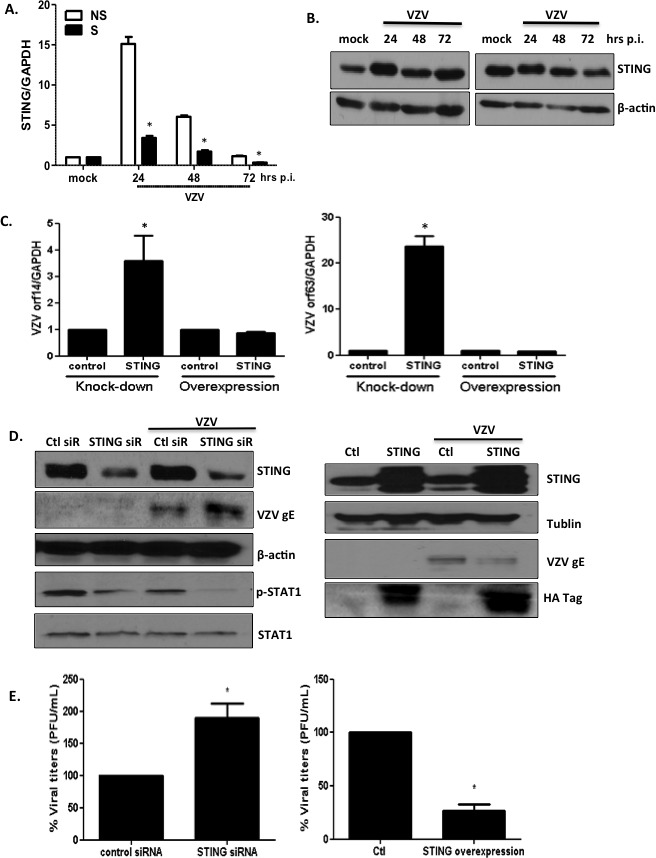
STING plays an important role in modulating replication of VZV (**A**) NS and S NHDFs were infected with VZV at an MOI of 0.001 for indicated time points. Host *STING* mRNA expression levels were measured by qPCR. The expression of *STING* was normalized to *GAPDH*. NS cells that were mock infected were arbitrarily set to 1. We performed qPCR assays in duplicate. The mean ± SD from three independent experiments is shown. *p < 0.05 vs. NS cells. (**B**) Western blotting detection of STING is shown. The blot shown is representative of results from three independent experiments. (**C**) MRC-5 cells were transfected with *STING*-specific siRNAs or STING-HA expression plasmids and infected with VZV at MOI 0.01. VZV ORF14 (late) and 63 (immediate early) gene expressions were measured by qPCR. Transcript expression levels were calculated in relation to the expression level of *GAPDH* mRNAs. We performed qPCR assays in duplicate, with the mean ± SD from all experiments shown. * *p* < 0.05 compared with control siRNA-transfected cells or control plasmid-transfected cells. (**D**) STING, pSTAT1, total STAT1, VZV gE were detected by western blotting (left: STING knockdown, right: STING overexpression). β-actin and tubulin were used as a loading control. Images shown are representative of results from three independent experiments. (**E**) Progeny VZV titers (PFU/mL) were calculated and presented as the mean ± SD from three different experiments, and as a percentage of control siRNAs. * *p* < 0.05 compared with control siRNA-transfected cells or control plasmid-transfected cells.

VZV is a DNA virus; additionally, RNA-seq analysis identified virus sensing pattern recognition receptors as the top upregulated networks in both progeria and NHDF cells. Therefore, we investigated the replication efficiency of VZV after knocking down the expression of the major dsDNA-sensing NLR: stimulator of interferon genes (STING) [25]. First, we attempted to determine whether VZV infection results in the elevation of the expression levels of *STING*. Both mRNA and protein levels of STING were upregulated on VZV infection in primary fibroblasts (Figure 8A and 8B). Interestingly, at 24 hpi, the level of induction of STING was higher in non-senescent cells than in senescent cells. In order to determine whether knockdown or overexpression of *STING* in MRC-5 cells affects the efficiency of viral replication, we transfected cells with *STING*-specific short interfering RNAs (siRNAs) or overexpressed HA-tagged STING plasmid prior to infection with VZV for 24 h. Overexpression of *STING* was confirmed by determining the expression levels of *STING* and HA for transfection efficiency. Silencing of STING expression led to a significant increase in the expression of VZV genes ORF14 and ORF63 (Figure 8C); however, similar levels of ORF14 and ORF63 were observed in cultures with overexpressed STING. Western blotting revealed a significant attenuation of STING protein expression following siRNA treatment. Increased gE expression was observed following STING knockdown in primary fibroblasts (Figure 8D). Additionally, investigation of the downstream effects of STING knockdown revealed that phosphorylation of STAT1, which signals expression of interferon-induced antiviral genes, was correlatively reduced. STING overexpression led to suppression of VZV gE synthesis. Furthermore, STING knockdown contributed to a 2-fold increase in VZV titers (*p* < 0.05) at 48 h post-transfection in primary fibroblasts, whereas STING overexpression resulted in a significant increase in VZV plaque numbers (Figure 8E). Our results indicate that the STING-mediated STAT1 antiviral pathway may be essential in controlling viral replication.

## DISCUSSION

The reactivation of VZV causes shingles, which is associated with significant morbidity, and sometimes, mortality in the elderly. Shingles potentially has a devastating impact on the patient's quality of life [26]. However, the role of immunosenescence during VZV infection is poorly understood. Moreover, currently available vaccines against VZV show poor efficacy in the elderly. In order to characterize the aspects of immunosenescence that contribute to increased VZV susceptibility, a clinical strain of VZV was isolated from a patient with shingles and used to infect two aging cell models: HGPS fibroblasts and replicative senescence-induced NHDFs. Using high throughput RNA-seq technology to comprehensively annotate host transcriptomes following infection, the present work illustrates the effects of immunosenescence in aging cells on viral replication efficiency and host signaling pathways.

VZV replication in skin cells is crucial for viral pathogenesis. Accordingly, we compared the transcription levels of major VZV genes, such as ORF14 and ORF 63, in aging skin cell models. Transcript levels of VZV immediate early gene (ORF63) in senescent cells (both stress-induced and replicative senescence) were found to be significantly higher than in non-senescent cells at 24 hpi. On the other hand, a relatively moderate induction of interferon signaling genes, such as IFN-β, was detected in senescent cells compared with non-senescent cells. This observation may reflect a close correlation between viral replication efficiency and host responses related to virus sensing and antiviral defense mechanisms. Given that the production of type I IFNs and the subsequent induction of IFN-stimulated genes (ISGs) is inhibited by various ORFs of VZV, such as ORF61, ORF62, and ORF47 [27-29], a detailed profile of dual viral and host gene expression patterns at various stages post-infection would provide a valuable insight into temporal changes in host/pathogen interaction.

The question of whether HGPS actually recapitulates the normal aging process at the cellular and organism levels has received much attention. Recent studies indicate that signaling pathway activation states in cells derived from chronologically young patients with HGPS strongly resemble those in cells from normal middle-aged and elderly individuals [10]. Mutations in the *LMNA* gene of patients with HGPS results in increased rates of cellular apoptosis, and consequently, a premature loss of functional “young” cells and concomitant premature accumulation of senescent cells [30, 31]. When we compared cellular morphology and proliferation patterns in fibroblasts derived from different age groups of patients with HGPS, we noticed that HG8 fibroblasts (isolated from an 8-year-old patient with HGPS) displayed a distinct cellular morphology, evidenced by maximal senescence-associated β-gal staining. In accordance with this data, VZV infection was found to induce an approximately 2-fold higher number of DEGs in HG8 fibroblasts than in HG3 and HG5 fibroblasts, as shown by Venn diagram and IPA analysis, although many of the DEGs identified were found to be unrelated to immune function (Figure 3). The unique cellular phenotype of HG8 fibroblasts potentially explains the low level of VZV replication, followed by the weak response to type I IFN, in these cells compared with HG3 and HG5 fibroblasts (Figures 1 and 7).

IPA analysis revealed that during the early stages of VZV infection, the triggering receptor expressed on myeloid cells 1 (TREM1) signaling pathway, PRRs of bacteria and viruses, IFN response, and p38 MAPK signaling are upregulated in both progeria cells and NHDFs infected with VZV. The TREM1 pathway, an essential regulator of antiviral immunity [32], was found to be the top predicted canonical pathway to be activated following B cell receptor-mediated reactivation of Epstein-Barr virus [33]. TREM1 is upregulated to a higher extent in HG5 cells than in HG3 or HG8 cells, whereas non-senescent NHDFs exhibit increased TREM1 expression than senescent NHDFs. Accordingly, in the present study, IPA analysis of VZV-infected senescent cells revealed that genes involved in virus-mediated inflammatory processes, such as TREM1 signaling, acute phase response signaling, and virus receptor signaling, were upregulated (Figures 3 and 5).

Age-dependent alterations in innate immune responses and interferon signaling have been noted in several studies [34]. In particular, delayed interferon production has been found to be associated with heightened susceptibility to viral infections such as those caused by the West Nile Virus (WNS) [35]. These data correlate well with the data presented in this study, in which lower production of interferon-β was observed in aging cells, in correlation with the abrogation of phosphorylation of STAT1 at later time points during infection (72 hpi). It has recently been shown that senescence is associated with the alteration of the expression patterns of a number of genes encoding secreted proteins such as cytokines and chemokines [36]. The production of these factors, collectively referred to as SASP factors, is an essential characteristic of senescent cells. Our findings indicate that the levels of SASP factors, such as IL-6, were robustly upregulated even in the mock-infected senescent NHDFs compared with non-senescent NHDFs. However, IL-6 levels did not change significantly over time in senescent NHDFs, but continued to increase in non-senescent NHDFs. Furthermore, the phosphorylation of p38 MAPK, which occurs as part of a major signaling pathway involved in the induction of senescence [37] and also upstream of the secretion of proinflammatory cytokines such as IL-6, was induced upon VZV infection. Consistent with the reduction in IL-6 protein levels in senescent NHDFs, phosphorylation of p38 MAPK was also attenuated in these cells in comparison with non-senescent NHDFs. Interestingly, levels of efficiency of viral replication were negatively correlated with levels of activation of host signaling pathways involved in interferon regulatory genes and SASP secretion, highlighting the possibility that age-related impairment of the innate immune response contributes to increased susceptibility to VZV infection and poor response to vaccination.

Another important finding of the current study was the identification of a novel VZV recognition receptor. To date, little information has been published regarding the potential receptors of VZV [1, 38]. Given that VZV possesses a DNA genome, it is highly likely that the intracellular sensing system of the host is important for VZV recognition. Recent studies indicate that STING is essential for activation of TANK-binding kinase 1 (TBK1) signaling pathways in response to herpes simplex virus-1 (HSV-1) infection. [39, 40]. In the present study, we demonstrate that siRNA-mediated silencing of STING and the subsequent abrogation of downstream molecule STAT1 in human fibroblasts leads to elevated viral titers (Figure 6). These findings suggest that STING also plays an important role in enhancing immunity against VZV infection. It is highly plausible that STING knockdown may have resulted in decreased antiviral signaling and subsequent reduction in interferon gene expression, potentially contributing to the induction of higher viral titers in VZV-infected cells. STING knockdown in Japanese encephalitis virus (JEV) infection model has also been found to result in increased intracellular viral load, whereas overexpression contributed to decreased viral titers [41]. Although STING is essential for IFN responses against herpes simplex virus [40], its role during VZV infection has not been previously demonstrated. STING-mediated IFN responses have been shown to be inhibited by age-enhanced endoplasmic reticulum stress during *Streptococcus pneumoniae* infection in an *in vivo* model [42]. We also compared the mRNA and protein expression levels of STING in non-senescent and senescent NHDFs, and found that senescence led to decreased STING mRNA expression at all time points (24, 48, and 72 hpi). As VZV is capable of evading innate immune activation, further investigation of the viral proteins that interact with STING would be of interest. Given that STING agonists have been considered potential adjuvant candidates, our study of STING as a potential receptor for VZV raises the possibility that a STING agonist may be useful as an adjuvant for vaccines against shingles.

Collectively, our data provide a comprehensive transcriptome analysis of aged human fibroblasts infected with VZV. Genes involved in several biological pathways related to innate antiviral immunity and inflammation were among the top DEGs. Differential expression of specific factors observed in aging cells may explain the observed variation in innate immune responses. These findings provide a framework for future studies examining the molecular mechanisms underlying the pathogenicity of VZV. Further global studies involving patients of different age groups with shingles should provide valuable insights into the efficient replication of VZV and suppression of the antiviral response in the elderly.

## MATERIALS AND METHODS

### Cells

Primary skin fibroblasts were obtained from the Progeria Research Foundation Cell and Tissue Bank (http://www.progeriaresearch.org/cell_tissue_bank.html). The HGPS cell lines HGADFN127, HGADFN122, and HGADFN167 were derived from 3-, 5-, and 8-year-old donors, respectively. Cells were maintained in Dulbecco's modified Eagle's medium (DMEM; Invitrogen, Carlsbad, CA, USA) supplemented with 15% fetal bovine serum (FBS), 2 mM L-glutamine, 50 units/mL penicillin, 50 μg/mL streptomycin, and 10 mM non-essential amino acids (Invitrogen) at 37°C/5% CO_2_. Normal fibroblasts typically grew for around 30 passages; however, HGPS fibroblasts were used before 10 passages, under the same culturing conditions.

NHDFs were purchased from Lonza, Basel, Switzerland. For stress-induced senescence, hydrogen peroxide (Sigma, St. Louis, MO, USA) was added at a concentration of 100 nM to cells in 6-well plates and incubated for 1 h before the addition of virus. For replication-induced senescence, NHDFs (Lonza, Basel, Switzerland) were grown as adherent cultures in fibroblast basal medium supplemented with FGM SingleQuots (Lonza). Non-senescent cells were defined as low-passage-number cells (less than 7 passages), whereas senescent cells were considered high-passage-number cells (more than 20 passages with positive β-galactosidase staining). MRC-5 cells (human lung fibroblasts) were obtained from ATCC (Manassas, VA, USA) and grown in DMEM (Lonza) with 10% HyClone FBS (GE Healthcare) and antibiotics.

### Senescence model

Stress-mediated senescence was induced by hydrogen peroxide (H_2_O_2_) treatment of non-senescent NHDFs. Senescence was validated as the percentage of senescence-associated β-galactosidase (SA-β-Gal) stained cells. For replicative senescence, NHDFs were allowed to grow for more than 20 passages, and senescent cells were verified by their delayed population-doubling times and through the use of a Senescence β-Galactosidase Staining Kit (Cell Signaling Technology, MA, USA), as described previously [22]. After staining, cells were washed and assessed by light microscopy (Olympus, Tokyo, Japan). For 4,6-diamidino-2-phenylindole (DAPI) staining, cells were grown in 24-well plates. After 24 h of incubation at 37°C and 5% CO_2_, cells were fixed with ice-cold 4% paraformaldehyde for 10 min and washed twice with PBS. Cells were then permeabilized with 100% methanol for 10 min, and coverslips with adherent cells were mounted onto glass slides using mounting media containing DAPI (Sigma) for visualization by confocal microscopy (Zeiss LSM700).

### VZV plaque assays

The previously described VZV strain YC01 (GenBank Accession No. KJ808816) (Won, Kim, Kim, & Lee, 2014) was cultured in human fetal fibroblasts (HFFs). Progeria fibroblasts or NHDF cells were infected with VZV at a MOI of 0.001 or 0.01. VZV-infected cells were diluted 10-fold and used to inoculate confluent uninfected HFF monolayers. After adsorption for 1 h, virus-containing media were removed and fresh media added to cultures. Cells were washed with media and incubated at 37°C and 5% CO_2_. After 3∼7 days, cells were fixed with 4% formaldehyde and stained with 0.03% crystal violet. Plaques were counted using a phase-contrast microscope.

### Gene expression analysis using RNA-seq

Total RNA from primary fibroblast cultures was isolated using TRIzol reagent (Invitrogen Life Technologies). The quality of all RNA samples was assessed using a Bioanalyzer 2100 (Agilent Technologies, Santa Clara, CA, USA). Generation of the RNA-seq library for each sample was carried out using the manufacturer's recommended protocols for an Illumina HiSeq2500 (Illumina, San Diego, CA, USA), to generate 101 bp paired-end reads. Reads that passed quality control were mapped to human IGenome (Ensembl_CRCh37) using Tophat2 (v2.0.13), and were counted using HTseq (v0.6.1p1).

### Identification of DEGs and functional analysis (GO, KEGG)

EdgeR (v3.4.2) was used to identify DEGs; this program used the Cox-Reid profile-adjusted likelihood method to estimate dispersion for pairwise comparisons. After negative binomial models are fitted and dispersion estimates are obtained, a generalized linear model (GLM) likelihood ratio test was used to determine differential expression. In this study, genes with ≥ 2-fold change and FDR-adjusted *q* value < 0.05 were considered significantly differentially expressed. Functional classification of genes was performed using the Database for Annotation, Visualization, and Integrated Discovery (DAVID) (http://david.abcc.ncifcrf.gov/). The representation of functional groups in each sample relative to the whole genome was investigated using the Expression Analysis Systematic Explorer (EASE) tool [25] within DAVID. The EASE tool uses a modified Fisher's exact test to measure enrichment of gene ontology (GO) terms. In order to identify enriched GO terms, functionally clustered genes were filtered using an EASE value less than 0.05 and selected. KEGG pathway analysis was also used for target gene candidates. Genes with FDR < 0.05 were considered as significantly enriched among target gene candidates.

### Ingenuity pathway analysis (IPA)

Data were analyzed using QIAGEN's Ingenuity Pathway Analysis (IPA) (QIAGEN Redwood City, USA). The most significant canonical pathways and functional processes of biological importance were selected using the list of DEGs identified by RNA-seq and the Ingenuity Pathways Knowledge Base. The Ingenuity Knowledge Base contains the largest database of manually curated and experimentally validated physical, transcriptional, and enzymatic molecular interactions. Furthermore, each interaction in the Ingenuity Knowledge Base is supported by previously published information. The list of DEGs was overlaid onto a global molecular network developed from information contained in the Ingenuity Pathways Knowledge Base, and networks were algorithmically generated on the basis of connectivity. Pathway enrichment p-values (Fisher's exact test) and activation z-scores were calculated by IPA.

### qPCR assays

Total RNA was isolated using TRIzol reagent (Life Technologies). First-strand synthesis of cDNA from total RNA was performed using ImProm-II™ Reverse Transcription System (Promega, Madison, WI, USA) according to the manufacturer's instructions. Quantitation of cDNA was performed by qPCR with SYBR Green PCR Master Mix (Life Technologies). Thermal cycling parameters consisted of an initial denaturation step at 95°C for 10 min, followed by 40 amplification cycles (95°C for 30 s, 60°C for 1 min). Primer sequences are available on request. The specificity of each reaction was confirmed by melting curve analysis and agarose gel electrophoresis of PCR products. The expression of target genes was normalized to that of human glyceraldehyde 3-phosphate dehydrogenase (GAPDH) and analyzed using the comparative Ct method.

### Enzyme-linked immunosorbent assays (ELISAs)

In order to determine the concentration of interleukin (IL)-6 in samples, we used a specific ELISA kit (BioLegend, San Diego, CA, USA) according to the manufacturer's instructions. Absorbance at 450 nm was determined using a microplate spectrophotometer. Protein concentrations were calculated based on standard curves generated using known concentrations of IL-6.

### Western blotting analysis

Cells were lysed at the specified times post-infection with lysis buffer (0.05 M Tris, pH 7.4, 0.15 M NaCl, 0.5 mM phenylmethylsulfonyl fluoride, 50 μg/mL aprotinin, 10 μg/mL leupeptin, 50 μg/mL pepstatin, 0.4 mM sodium orthovanadate, 10 mM NaF, and 10 mM sodium pyrophosphate). Lysates were resolved by sodium dodecyl sulfate polyacrylamide gel electrophoresis (SDS-PAGE) on 10-12% acrylamide gels. Proteins were transferred to polyvinylidene difluoride (PVDF) membranes, and blocked with 5% (w/v) skim milk in Tris-buffered saline (0.2 M Tris, 1.36 M NaCl) supplemented with 0.1% (v/v) Tween-20 (TBS-T) for 1 h at room temperature. Membranes were washed with 15 mL of TBS-T and then incubated with primary antibody for 1 h at 25°C. After further washing with TBS-T, membranes were incubated with the appropriate horseradish peroxidase (HRP)-conjugated antibody for 1 h at 25°C. Membranes were washed with TBS-T and incubated with Pierce ECL Western blotting substrate kit (Thermo Scientific), and exposed to film. We used a mouse anti-VZV gE antibody (ab52549; Abcam) diluted 1:10,000 to detect major VZV glycoproteins, followed by an anti-mouse HRP-conjugated antibody (1:5,000; Invitrogen). Antibodies against HA, phospho-STAT1, STAT1, and STING were purchased from Cell Signaling Technology, and anti-β-actin (Abgent), anti-tubulin (Abgent), and anti-GAPDH (Sigma) antibodies were used as internal controls.

### Overexpression and knockdown experiments

MRC-5 cells were seeded in 6-well plates and allowed to grow until cultures were 70% confluent on the day of transfection. Transient transfections with either scrambled control or *STING*-specific siRNAs (Bioneer, Daejeon, Korea) were performed using Lipofectamine^®^ (Invitrogen), according to the manufacturer's protocol. Briefly, 1 μL of *STING* siRNA and 5 μL of Lipofectamine^®^ were mixed with 100 μL of Opti-MEM^®^ (Invitrogen). The mixture was incubated for 15 min at room temperature and then added dropwise to each culture well containing 1 mL of Opti-MEM^®.^ At 6 h post-transfection, transfection medium was aspirated and fresh growth medium was added. At 24 h post-transfection, cells were infected with VZV strain YC01 at an MOI of 0.01. Cells were cultured for 24 h, lysed, and lysates subjected to western blotting to determine *STING* expression levels. For overexpression of *STING*, 4 μg of STING-pUNO-HA plasmid (Invivogen) was transfected using InFECT transfection reagent (iNtRON Biotechnology, Korea) according to the manufacturer's instructions.

### Statistical analysis

Results were expressed as the means ± standard deviation (SD). Data were analyzed using Student's t-test to determine statistically significant differences between two groups. Statistical analyses were performed using GraphPad Prism (GraphPad Software, La Jolla, CA, USA). A *p*-value of < 0.05 was considered statistically significant.
